# Elastic Band Training Across Competitive Levels in Female Soccer: A Randomized Controlled Trial With Follow-up

**DOI:** 10.1177/19417381261490002

**Published:** 2026-10-12

**Authors:** Henrique Sousa, Eduardo Abade, Júlio A. Costa, João Barreira, Aarón Miralles-Iborra, Filipe Maia, Rui Marcelino

**Affiliations:** † Research Center in Sports Sciences, Health Sciences and Human Development, CIDESD, ELITE Research Community, Vila Real, Portugal; ‡ FPF Academy, Portuguese Football Federation, Oeiras, Portugal; § University of Maia, Maia, Portugal; ¶ Department of Sports Science, Exercise and Health, University of Trás-os-Montes e Alto Douro, UTAD, Vila Real, Portugal; ‖ Sports Research Centre (Department of Sport Sciences), Miguel Hernandez University of Elche, Alicante, Spain; # Polytechnic Institute of Maia Research Center (N2i), Maia, Portugal

**Keywords:** adult athletes, resistance bands, soccer, women, youth athletes

## Abstract

**Background:**

Resistance training is a fundamental component of performance in female soccer; however, conventional weight training may be impractical for teams with limited facilities and equipment. Elastic band resistance training (EBT) offers a practical alternative.

**Hypothesis:**

A 9-week EBT program will enhance lower-limb strength, vertical jump, and change-of-direction (COD) speed in trained and highly trained female soccer players.

**Study Design:**

Randomized controlled trial.

**Level of Evidence:**

Level 2.

**Methods:**

A total of 74 players were divided into Under-15 (U15; n = 25), Under-19 (U19; n = 25), and Senior (n = 24) groups. Each age group was assigned randomly to an experimental or control group. The intervention was integrated into the in-season regimen, performed twice weekly before training sessions, over 9 weeks. The training protocol included 6 resistance exercises split across 2 weekly circuit-based sessions. Isometric knee extension (KE) and knee flexion (KF) strength, countermovement jump (CMJ) height and power, and T-test performance were evaluated pre- and postintervention and after a 3-week detraining period.

**Results:**

Difference-in-differences ordinary least squares regression analysis revealed improvements in U19 KE strength of the dominant and nondominant leg (coefficient = 132.90 N; *P* = 0.03 and 131.59 N; *P* = 0.05, respectively). KF strength improved marginally in U15 and Senior groups. Senior players also showed a nonsignificant positive trend towards CMJ power (coefficient = 581.08 W; *P* = 0.09), with no relevant changes in CMJ height or T-test. After detraining, strength gains declined across all groups, while CMJ and T-test metrics remained stable.

**Conclusion:**

EBT enhances knee strength effectively, particularly in older age groups, but its influence on vertical jump and COD speed is limited. The reversal of strength gains after short-term detraining highlights the importance of consistent training for sustained athletic development.

**Clinical Relevance:**

EBT is a practical, low-cost method to improve lower-limb muscle strength in female soccer players and can be integrated easily into in-season training when traditional equipment is unavailable.

Soccer is an intermittent team sport that requires a combination of physical, physiological, technical and/or biomechanical, and tactical factors.^43^ As the popularity and professionalism of women’s soccer continues to rise globally, there is a growing interest to understand and optimize the specific athletic capacities required to perform effectively within the game’s demanding physical profile.^22^ Despite this trend, most of the scientific literature remains male-dominant, and research focusing on the training responses in female soccer players is still emerging.^2^ Variations in anatomical structure, physiological function, and hormonal profiles underscore the fact that findings from male athletes may not be directly applicable to female players.^37^ Specifically, female-specific neuromuscular recruitment patterns and the influence of estrogen on musculoskeletal stiffness requires specific loading strategies to optimize power output and mitigate injury risk.^37^ This gap presents an important opportunity to investigate evidence-based training methods that are both accessible and effective for performance optimization in female players.

Resistance training has been recognized as an integral component in sports performance, with well-established benefits in muscular strength, power, and hypertrophy, while also contributing to injury prevention.^44^ Although proven effective, traditional resistance training methods involving external loads (e.g., free weights or machines) may not be a practical or feasible option for all teams, particularly at youth and amateur levels where facilities and equipment are limited. In this context, elastic band resistance training (EBT) has gained considerable attention as a versatile, cost-effective, space-efficient, and portable alternative,^17^ suited specifically for these resource-constrained environments. Researchers have been investigating the effectiveness and practical applications of EBT among athletes of different ages and backgrounds. A recent systematic review presented positive results from EBT on the enhancement of various physical performance metrics, such as sprint speed, change-of-direction (COD) ability, and lower- and upper-limb strength and power in male and female athletes from various age groups and type of sports.^41^ However, there was no information available on female soccer players. Considering the sport’s reliance on explosive movements and physical contact, understanding how female players respond to EBT is key for developing training strategies that reflect the demands of soccer and the characteristics of female players.

Youth athletes undergo rapid growth and neuromuscular adaptations that may influence how they respond to a particular resistance training stimulus compared with their senior counterparts.^30^ Maturation, coordination, and previous training experience all play a role in shaping these responses, influencing how athletes adapt to a specific type of training.^28^ Understanding these differences is of critical importance for designing age-appropriate training programs that optimize performance outcomes. Therefore, evaluating the impact of EBT on physical performance across these stages of development is of theoretical and practical importance. Furthermore, periods of training cessation, commonly known as detraining, can lead to a reversal of physiological adaptations, compromising athletic performance.^32^ Investigating how players respond to both training and detraining phases is key to develop strategies that minimize performance loss and support long-term athletic development.

The present study aims to assess the effect of a 9-week, in-season EBT intervention on key physical performance metrics in trained and highly trained female soccer players among the Under-15 (U15), Under-19 (U19), and senior categories, followed by a 3-week postintervention follow-up evaluation (detraining).^27^ The performance metrics evaluated include knee extension (KE) and knee flexion (KF) maximal isometric strength, vertical jump height and power, and COD speed performance. Given the developmental differences between age groups, it is hypothesized that the magnitude of training responses might vary across players at different stages of maturation. By studying female soccer players across 3 distinct developmental stages, this research addresses a critical gap in the literature and provides practical insights into how EBT can be adapted to meet the specific performance needs of athletes at different ages.

## Methods

### Study Design

The present study utilized a randomized controlled trial with parallel-group design to investigate the effects of EBT on various performance outcomes in trained and highly trained female soccer players. Participants were randomized within their age cohort to either an experimental group (EG), which received the EBT intervention in addition to their standard training regimen, or a control group (CG), which continued doing only their standard training regimen. The intervention protocol was implemented over a 9-week period during the second half of the in-season, with physical performance tests at baseline and at postintervention. A follow-up assessment was also conducted 3 weeks after the postintervention testing, to identify the possible detraining effect when the EBT intervention was withdrawn from the EG. Figure 1 illustrates the study procedures and timeline.

**Figure 1.**
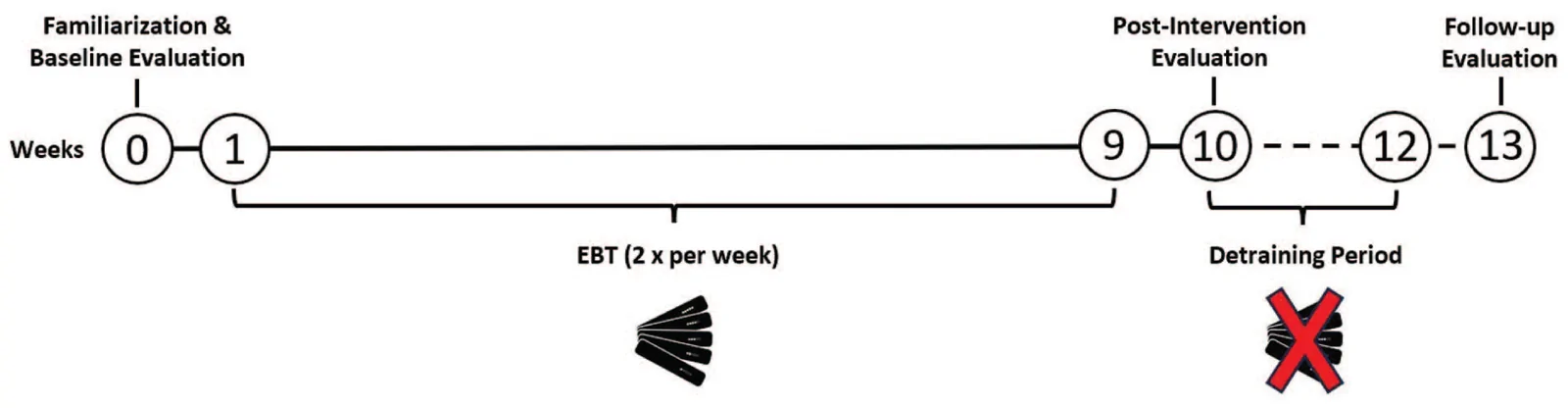
Study procedures and timeline for the EG. Numbers inside the circles indicate the study weeks. EBT, elastic band resistance training; EG, experimental group.

### Participants

An a priori power analysis was conducted using G*Power (Version 3.1) to determine the minimum sample size required for each age cohort, given that the EG and CG were compared independently within each cohort. The analysis was based on a 2-tailed *t* test with an effect size = 1.58, alpha level = 0.05, and power = 0.95. The effect size was derived from postintervention vertical jump height mean differences reported in similar literature involving youth female athletes.^16^ The result indicated that a minimum of 24 participants (12 per group: experimental and control) would be required to detect a significant difference between groups. To account for potential dropouts or exclusions, a larger sample size was initially recruited. Participants were eligible for inclusion if they met the following criteria: (1) actively training and playing in an amateur female soccer club in the U15, U19, or Senior age group; (2) competing at a Tier 2 (trained/developmental) or Tier 3 (highly trained/national) level, as defined by McKay et al^27^; (3) in good physical condition, without any injury at the time of recruitment or lingering effects from previous injuries that could compromise their performance or participation. Players were recruited from 2 amateur soccer clubs based in Portugal, competing at a similar level. All participants trained 3 times per week (~210 minutes of weekly training), with regular matches on weekends. U15 players competed at the regional level, whereas U19 and Senior players took part in national-level competitions. Following the intervention protocol, the sample selected for analysis consisted of 25 U15 players, 25 U19 players, and 24 Senior players. At follow-up, 23 U15 players, 22 U19 players, and 21 Senior players remained and were included in the final analysis. A CONSORT flowchart summarizing the recruitment, allocation and retention of participants is presented in Figure 2 and age groups descriptive statistics are exhibited in Table 1. This study was approved by the University of Maia Ethics and Deontology Board Ethics Committee (approval no. 155/2023). All participants and, when necessary, their legal guardians were informed of the study’s benefits and risks and provided written informed consent. The study protocol was registered on ClinicalTrials.gov (no. NCT06800079).

**Figure 2.**
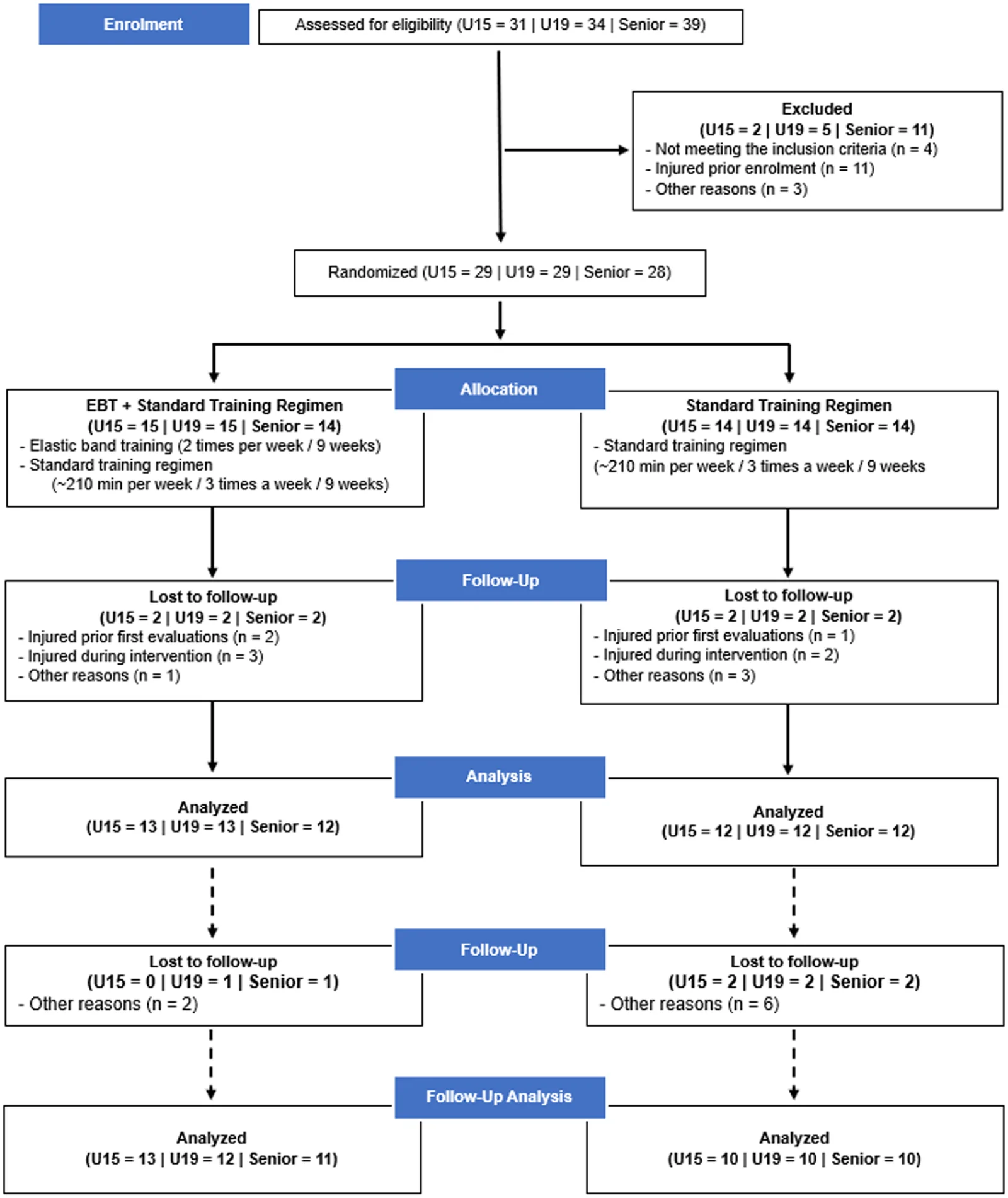
CONSORT flow diagram detailing recruitment, randomization, dropouts, and final sample size for the postintervention and the follow-up, across both groups. CONSORT, Consolidated Standards of Reporting Trials; EBT, elastic band resistance training; U15, under 15 years; U19, under 19 years.

**Table 1.** Age group descriptive statistics

	Participants	N	Age, years	Height, cm	Weight, kg	BMI, kg/m^2^
Postintervention	U15	25	13.4 ± 0.9	159.1 ± 6.6	56.1 ± 11.1	22.1 ± 3.9
	U19	25	17.1 ± 1.1	163.7 ± 4.8	62.0 ± 7.3	23.1 ± 2.6
	Senior	24	21.0 ± 2.5	163.3 ± 6.0	66.4 ± 10.3	25.0 ± 4.0
Follow-up	U15	23	13.4 ± 0.9	158.7 ± 6.5	55.0 ± 10.5	21.8 ± 3.7
	U19	22	16.9 ± 1.0	164.4 ± 4.5	61.7 ± 7.7	22.8 ± 2.6
	Senior	21	20.7 ± 2.4	163.2 ± 6.1	67.0 ± 9.4	25.2 ± 3.7

Data given as mean ± SD. BMI, body mass index; U15, under 15 years; U19, under 19 years.

### Testing Procedures

All participants first completed a familiarization session to ensure proper understanding and execution of each testing protocol. Before the evaluations, players performed a standardized warm-up consisting of bodyweight squats, dynamic stretching, sprint drills, and COD exercises. Each test consisted of 2 trials; a third trial was conducted when the evaluator judged the initial results to be inconsistent. The best score from the attempts was retained for analysis.^1^ A rest interval of 5 minutes was provided between each test to minimize fatigue effects.

### KE and KF Strength

Maximal isometric KE and KF isometric strength were evaluated by using a handheld dynamometer (DynaMo Plus, VALD Performance).^35^ Both dominant and nondominant legs were tested with measures categorized into KE dominant (KED), KE nondominant (KEND), KF dominant (KFD), and KF nondominant (KFND). KE was performed in a seated position and KF in a prone position. Using goniometry, the dynamometer chain was adjusted to position the knee at ~60° for extension and ~30° for flexion to ensure maximal torque generation.^21^ Each maximal voluntary isometric contraction was sustained for 5 seconds without any compensatory body movements. Players initiated their next attempt when they felt adequately recovered, and the highest scores were used for subsequent analysis.

### Countermovement Jump

Countermovement jump (CMJ) height and power were evaluated using a force platform system (ForceDecks, VALD Performance).^6^ Players started from an upright position, performed a rapid downward movement to ~90° of KF, and immediately propelled themselves vertically into the air. Players performed their next attempt when they felt rested, and the best results were used for analysis.

### T-Test

The Modified T-Test was applied to evaluate COD speed ability,^29^ involving directional changes such as forward sprinting, left and right-side shuffling, and backpedaling. Timing was recorded using photocells (WhittyGate, Microgate). Players were instructed to complete the test as fast as possible, with a 1-minute rest between attempts. The fastest time was used for analysis.

### Training Program

After the protocol familiarization and baseline measurement week, players underwent 9 weeks of EBT, with 2 sessions per week. All athletes in the EG completed all 18 EBT sessions. No participants, from either group, performed additional resistance training before the intervention and were instructed to continue doing so throughout the intervention. The training structure is presented in Table 2. The training program was performed 30 minutes before the beginning of the main practice on the pitch. Each session was preceded by a brief warm-up consisting of bodyweight squats to activate the lower-body musculature. The first weekly session (EBT1) consisted of glute bridges, hip abduction, and hip adduction, whereas the second session (EBT2) included Romanian deadlifts, hip flexion, and trunk rotation. These exercises were chosen due to their practicality and feasibility for implementation, as they require minimal equipment and can be integrated into players’ regular routines, including on-field sessions. Moreover, they are relevant for soccer players, targeting key muscle groups that are essential for both performance optimization and injury prevention.^9^ Each session followed a circuit format consisting of 3 sets, each comprising a series of exercises performed consecutively (Set 1: Ex1 + Ex2 + Ex3; Set 2: Ex1 + Ex2 + Ex3; Set 3: Ex1 + Ex2 + Ex3). Players rested for up to 30 seconds between exercises. All sessions were supervised directly by the investigators, who also monitored and corrected exercise technique (presented in Appendix A in the Supplementary Material, available in the online version of this article).

**Table 2.** EBT program performed by the EG over the 9-week protocol

	Exercise	Sets × Reps	RPE/RIR
EBT1	Glute bridges	3 × 8-10	8/2
	Hip abduction		
	Hip adduction		
EBT2	Romanian deadlift	3 × 8-10	8/2
	Hip flexion		
	Trunk rotation		

See Appendix A (available online) for exercise instructions. EBT, elastic band resistance training; EG, experimental group; RIR, repetitions in reserve; RPE, rate of perceived exertion.

Elastic resistance was provided through the use of Elastibands (Sveltus). These bands feature 8 numbered loops for standardized hand or foot placement positions and adjustable resistance. Three resistance levels were used for this study: 10 kg, 15 kg, and 20 kg, each distinguished by color. Following the framework described by Fredriksen et al,^12^ training intensity was determined by an 8 to 10 repetition maximum (eccentric/concentric = 3/1 seconds) with 2 repetitions in reserve (2RIR), corresponding to a rating of 8 on the rate of perceived exertion (RPE)/RIR scale.^49^ This approach aligns with current evidence showing that moderate repetition ranges combined with a controlled eccentric phase maximizes the hypertrophy stimulus effectively, which consequently enhances strength gains.^3^ During the familiarization session, players were instructed to select an elastic band based on this guideline and advised to increase resistance if they were able to perform >12 repetitions, with more than 2RIR. Resistance progression was individualized according to each player’s subjective RIR. Players who could complete >12 repetitions with >2RIR advanced to the next resistance level, monitored by the investigators at each session. Load was increased by using a stronger resistance band (indicated by color), changing the grip or foot position using the numbered slots, or adding a second band. Immediately after each session, players rated their perceived muscle soreness using a 0 to 100 mm visual analogue scale (VAS), where 0 indicated “absolutely no soreness” and 100 represented “the most muscle soreness they could possibly bear.”^24^

### Statistical Analyses

A difference-in-differences (DiD) framework was conducted to assess the effectiveness of EBT on several physical performance parameters.^5,38^ This method provides a reliable way to estimate causal effects by analyzing how outcomes evolve over time in the EG relative to the CG, before and after the intervention. In sports science, the DiD approach is particularly robust as it isolates the EBT intervention’s effects by controlling external factors, such as natural maturation in youth athletes or the accumulated fatigue of a competitive season, that may affect both groups simultaneously. The statistical analyses were conducted in Python using the statsmodels library, with robust standard errors used to account for heteroscedasticity. Based on these estimates, 95% CIs were calculated to assess the precision of the effects. The data were modeled using the following DiD specification:

$$\begin{array}{l} Y_{it}=\alpha +\beta_{1}\times Treatment_{i}+\beta_{2}\times Post_{t}\\ \;\;\;\;+\beta_{3}\times (Treatment_{i}\times Post_{t})+\varepsilon_{it}, \end{array}$$

where *Y_it_* represents the outcomes for participant *i* at time *t*, Treatment_*i*_ is a binary indicator denoting whether the player is in the EG (1 = treatment group, 0 = CG), Post_*t*_ is a binary indicator representing the post-treatment period (1 = post, 0 = pre), Treatment_*i*_ × Post_*t*_ is the interaction term, capturing the DiD estimator (i.e., the causal effect of the intervention), and *ϵ_it_* is an error term.^25^ Two distinct ordinary least squares (OLS) regressions were used for each of the 7 dependent variables (KEND, KED, KFND, KFD, CMJ height, CMJ power, T-test). The first model evaluated the efficacy of the EBT program by comparing pre- and postintervention results, while the second model examined the possible detraining effects by comparing the postintervention with the follow-up. These analyses provided insights into the impact of the intervention and the subsequent retention of its effects after its cessation. Test-retest reliability was assessed using the intraclass correlation coefficient (ICC) and coefficient of variation (CV).

## Results

Figure 3 summarizes the key findings from the DiD OLS regression models across 4 performance metrics: KE strength, KF strength, vertical jump, and COD speed. The results are presented as forest plots, which display the estimated DiD coefficient together with 95% CIs and corresponding *P* values for each age group. The models evaluated the effects of a 9-week EBT protocol and a subsequent 3-week detraining period. Refer to Table S1 and Table S2 in the Supplementary Material (available online) for a more complete overview.

**Figure 3.**
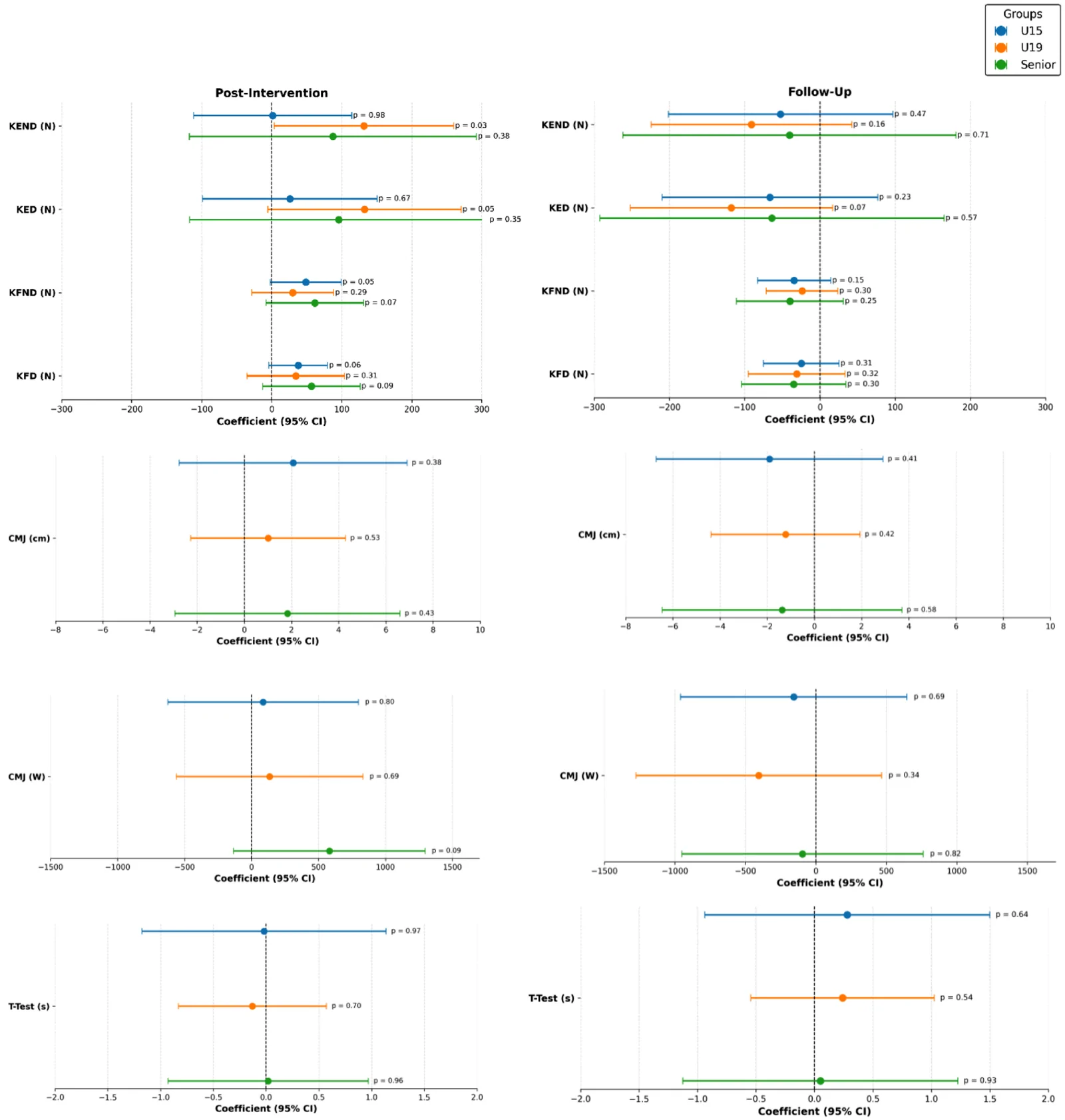
Forest plots displaying DiD coefficients (with 95% CIs) and *P* values for group comparisons across postintervention (left) and follow-up (right) timepoints. Each point represents the estimated coefficient for the respective age group, with error bars indicating 95% CI. CMJ, countermovement jump; DID, difference-in-differences; KE, knee extension; KED, KE of dominant leg; KEND, KE of nondominant leg; KF, knee flexion; KFD, KF of dominant leg; KFND, KF of nondominant leg; U15, under 15 years; U19, under 19 years.

### KE Strength

The DiD analyses showed that the U19 group experienced the largest intervention effects on KE strength, with increases of 131.59 N (*P* = 0.03) in the nondominant leg and 132.30 N (*P* = 0.05) in the dominant leg. The coefficient for the senior group (87.33 N, *P* = 0.38), while not statistically significant, suggests a potential positive effect on nondominant leg strength, with a comparable pattern in the dominant leg (95.75 N, *P* = 0.35). The U15 group showed limited improvement, with nonsignificant mean differences of 1.28 N (*P* = 0.98) in the nondominant leg and 25.92 N (*P* = 0.67) in the dominant leg. After the detraining period, reductions in KE strength were observed in all groups. Regarding the U19 players, the DiD estimates indicated decreases of −90.97 N (*P* = 0.16) in the nondominant leg and −117.70 N (*P* = 0.07) in the dominant leg. Among the senior players, extension strength declined −40.53 N (*P* = 0.71) in the nondominant leg and −63.80 N (*P* = 0.57) in the dominant leg. In the U15 group, the decreases were −52.27 N (*P* = 0.47) and −66.46 N (*P* = 0.23) for the nondominant and dominant legs, respectively.

### KF Strength

For the U15 players, the intervention effects approached statistical significance, reaching 48.53 N (*P* = 0.05) in the nondominant leg and 37.74 N (*P* = 0.06) in the dominant leg. The senior group similarly showed notable gains, though not statistically significant, with increases of 61.50 N (*P* = 0.07) and 56.67 N (*P* = 0.09) for the nondominant and dominant legs, respectively. In contrast, the U19 players exhibited smaller, nonsignificant effects of 29.84 N (*P* = 0.29) in the nondominant leg and 34.38 N (*P* = 0.31) in the dominant leg. After detraining, KF strength declined in all groups. In the U15 players, decreases were shown to be −34.31 N (*P* = 0.15) in the nondominant and −24.88 N (*P* = 0.31) in the dominant leg. The senior group recorded reductions of −39.95 N (*P* = 0.25) and −34.87 N (*P* = 0.30), while the U19 players declined by −23.85 N (*P* = 0.30) and −30.80 N (*P* = 0.32) in the nondominant and dominant legs, respectively.

### Vertical Jump

The coefficients for jump height were small and not statistically significant (U15, 2.07 cm; *P* = 0.38; Senior, 1.83 cm; *P* = 0.43; U19, 1.01 cm; *P* = 0.53), offering limited evidence of an intervention effect. For jump power, the senior group showed the largest point estimate, although nonsignificant, with an increase of 581.08 W (*P* = 0.09). In comparison, U19 players had nonsignificant improvements by 135.46 W (*P* = 0.69), and U15 players by 87.10 W (*P* = 0.80). After the detraining period, CMJ performance declined slightly in all groups. The U15 players registered a decrease of −1.90 cm (*P* = 0.41), U19 players declined −1.22 cm (*P* = 0.42), and senior players dropped −1.37 cm (*P* = 0.58). In terms of jump power, the most pronounced decrease occurred in the U19 group, with a reduction of −405.15 W (*P* = 0.34). U15 players showed a smaller decline of −157.60 W (*P* = 0.69), while senior players experienced only a minor decrease of −95.39 W (*P* = 0.82).

### COD Speed

The postintervention analyses exhibited minimal, nonsignificant changes across all age groups. The U15 group became faster by −0.02 seconds (*P* = 0.97), U19 players by −0.13 seconds (*P* = 0.70), while senior players were 0.02 seconds slower (*P* = 0.96). After the detraining period, U15 players became slower by 0.28 seconds (*P* = 0.64), U19 players by 0.24 seconds (*P* = 0.54), and senior players by 0.05 seconds (*P* = 0.93).

### Reliability Analyses

Table 3 illustrates performance data reliability outcomes for the evaluations that were conducted. ICCs showed acceptable reliability, ranging between 0.771 and 0.945, with CVs ranging between 2.07 and 9.91.

**Table 3.** Test-retest reliability of the applied evaluations

	ICC (*P* value)	CV, %
KEND	0.781 (<0.001)	7.72
KED	0.771 (<0.001)	9.91
KFND	0.901 (=0.009)	7.31
KFD	0.809 (=0.01)	8.65
CMJ, cm	0.929 (<0.001)	3.74
CMJ, W	0.945 (<0.001)	2.60
T-test	0.872 (=0.006)	2.07

CMJ: countermovement jump; CV, coefficient of variation; ICC, intraclass correlation coefficient; KE, knee extension; KED, KE of dominant leg; KEND, KE of nondominant leg; KF, knee flexion; KFD, KF of dominant leg; KFND, KF of nondominant leg.

## Discussion

This research aimed to investigate the effects of a 9-week EBT protocol on lower-limb strength, vertical jump, and COD speed performance in trained and highly trained female soccer players across 3 age groups (U15, U19, and Senior). Furthermore, it also explores how these adaptations changed after a 3-week detraining period.

In regards to KE strength, U19 players who completed the EBT program demonstrated a significant increase in KE strength compared with their control counterparts. These estimates were accompanied by relatively low standard error (Table S1, available online), which supports the consistency of the training effect and is reflected in the statistically significant outcomes. In this context, the *P* values help confirm that the observed differences are unlikely to be due to chance, given the model’s assumptions. Senior players also showed increases in KE after the EBT intervention; however, the SE in this group were larger than the coefficient (Table S1, available online), indicating greater variability in individual responses. This variability is reflected in higher, statistically nonsignificant, *P* values, which suggests less certainty in the group-wide effect (Table S1, available online). Still, the magnitude of the gains observed in the EG relative to CG suggests a potentially meaningful training effect, despite the lack of statistical significance. Conversely, U15 players presented only minimal, nonsignificant improvements. These minimal changes suggest that the EBT stimulus may have been insufficient for this group. It can be hypothesized that these results are attributed to the fact that none of the exercises that were included in the training program directly targeted the quadriceps - the primary muscle group responsible for KE.^31^ The positive results from the U19 and Senior players are likely due to greater neuromuscular maturity, which enhances motor unit recruitment and intermuscular coordination, thus improving the transfer of strength gains from indirectly targeted exercises.^39,45^ Beyond exercise selection, the attenuated response in U15 players may also reflect their maturity status. In pubertal athletes, rapid growth is accompanied by ongoing changes in muscle-tendon properties, motor control, and coordination, which can constrain short-term gains in maximal strength for a given training dose.^45^ Consequently, while the present EBT protocol was sufficient to elicit clear KE gains in older, more mature players, younger athletes may require a longer intervention, higher volume, or more targeted quadriceps loading to achieve comparable improvements.^23^

KF strength also showed positive developments. U15 players who followed the EBT protocol demonstrated larger improvements compared with the CG, which exhibited minimal change or even decline over time, as indicated by the Period coefficient (Table S1, available online). It needs to be stated, however, that there was a pre-existing difference between groups at baseline in KFD, as shown by the Treatment coefficient. Such imbalance can affect internal validity because it may bias simple between-group comparisons. However, the DiD approach helps address this issue by evaluating relative changes over time rather than absolute differences, which preserves said validity. Still, the baseline discrepancy should be considered when interpreting the magnitude of the observed effects. Nonetheless, the DiD coefficient revealed a positive development in the EG, suggesting that EBT had a reinforcing effect on KF strength in this age group. Senior players responded similarly, with improvements that exceeded those of the CG, which showed a downward trend over time, again reflected in the Period coefficient. Despite moderate variability in individual responses and *P* values slightly above conventional thresholds, the direction and magnitude of the DiD coefficients support a potentially relevant training effect. These results are in line with previous research,^30,48^ and highlight the importance of knee flexors in female soccer players, particularly in reducing the risk of hamstring and anterior cruciate ligament injuries,^34^ although this connection is inferred from existing literature rather than measured directly in this study. Curiously, more complex outcomes were discovered in U19 players. Gains were smaller and nonsignificant, reflecting greater uncertainty; however, the Period coefficient of both KF metrics suggests that CG experienced a slight decline over time, contrasting with the positive changes in EG. This may indicate that EBT helped counteract a decline in muscle strength, possibly related to accumulated fatigue during the season,^19^ and can assist in neuromuscular performance preservation. This corroborates with previous research that states that resistance training may help prevent performance loss due to fatigue by improving muscle strength.^10,13,20^

After the detraining period, strength adaptations declined in both KE and KF, with some differences between age and muscle groups (Table S2, available online), although none of these changes reached statistical significance. U19 players showed the largest point-estimate declines in KE, followed by U15 players, while Seniors showed the smallest losses. A possible explanation is that Senior players likely retained more strength due to neuromuscular consolidation, which stabilized the strength adaptations against the acute losses seen in the younger cohorts.^14,32^ Despite these reductions, the EG likely remained stronger than the CG, as the initial advantage was not fully erased. Interestingly, the Period coefficient for U15 KEND was 29.50 N, indicating that the CG improved slightly during the detraining phase. This contrasts with the general trend of decline seen in the other groups and metrics, possibly indicating natural recovery or reduced fatigue. Furthermore, KF strength appeared more resilient to detraining, with all age groups experiencing declines (Table S2, available online). Notably, the Period coefficient for U15 KFND was 29.00 N, indicating that the CG improved slightly during detraining. The slight improvements in the nondominant leg strength during the detraining phase may reflect the dissipation of cumulative fatigue and lower match or training load, allowing neuromuscular recovery and a more accurate assessment of baseline strength.^40^ Nevertheless, this reduction in performance highlights the reversible nature of neuromuscular adaptations after resistance training cessation,^4^ emphasizing the importance of program consistency to maintain progress. Furthermore, training intensity seems to be a key component to ensure strength gains retention during periods of reduced training volume.^42^

Vertical jump height showed minimal, nonsignificant improvements across all groups. This suggests a potential benefit from the training, yet the high variability limits confidence in the effect. Senior players showed the largest improvement, as reflected in the DiD coefficient (Table S1, available online). However, this gain came with a higher SE and a *P* value above conventional significance thresholds, indicating considerable variability and limited statistical certainty. These results contrast with those of Hammami et al,^15^ who reported significant improvements in CMJ height after a 10-week complex strength training using elastic bands in young female handball players. The discrepant results found in this study could be explained by the intervention. Although the training period was similar in both investigations, the intervention of the present study did not incorporate plyometric exercises. This type of training prepares muscles and tendons to store and release elastic energy more effectively during the stretch-shortening cycle,^26,46^ potentially explaining the differing results. Moreover, as previously mentioned, the absence of quadriceps-targeted exercises may have further attenuated improvements in vertical jump height, given its importance in generating KE torque.^18^

Vertical jump power exhibited mixed results, with the most promising outcomes observed in Senior players. In both U15 and U19 groups, CMJ power increased after the intervention, as reflected in the DiD coefficients (Table S1, available online). However, the CG also showed substantial gains over time, suggesting that the training did not outperform natural development. The high standard error and lack of statistical significance further limit the interpretability of these effects. In contrast, Senior players demonstrated notable improvements, while the CG declined over time, as indicated by the Period coefficient. This pattern suggests that EBT may have helped counteract losses in explosive performance. This raises the possibility that the observed results may be attributed to age and maturity differences between groups. Previous research suggests that biological maturation in girls ends at 17 years of age and tends to plateau as athletes approach neuromuscular maturity.^7,8,36^ Therefore, Senior players may have demonstrated a more developed neuromuscular system, which possibly contributed to their marginal statistical improvements in vertical jump power despite the absence of plyometric exercises.

After the detraining period, both vertical jump metrics decline across all ages, reversing the small improvements observed during the intervention, though none of the detraining-phase changes reached statistical significance. During the follow-up, all groups experienced small declines in height (Table S2, available online). Moreover, the Period coefficients were close to zero, suggesting minimal change in the CG. Regarding vertical jump power, the U19 EG experienced a sharp decline (–405.15 W), while CG improved (407.40 W), suggesting a complete reversal of the training effect and resulting in the EG falling below the CG by the end of the washout period. In U15 players, the EG also regressed, whereas the CG showed only a modest decline, indicating that the EG lost more than the CG and failed to retain the gains achieved during training. Among Senior players, the EG declined during detraining, while the CG showed a modest improvement (38.30 W). Despite this shift, the EG had started the detraining phase with a performance advantage (387.86 W), suggesting that they likely remained superior in absolute terms. This pattern indicates that older athletes may retain more of their explosive capacity after short-term detraining, potentially due to more consolidated neuromuscular adaptations.

COD speed performance did not show significant improvements in any of the age groups (Table S1, available online). This supports the idea that while the EBT and other types of resistance training can help develop general strength, improvements in COD speed are also highly dependent on straight sprint and technique training, where foot placement, adjustment of stride to accelerate and decelerate and body lean and posture are key physical determinants when executing rapid COD.^33,47^ In addition, multiplanar plyometric training has also been shown to be beneficial for improving COD ability,^11^ emphasizing the need for the inclusion of this type of training in future interventions. After detraining, COD speed remained unaffected, as DiD coefficients were minimal and statistically nonsignificant across all age groups, and neither Treatment nor Period coefficients revealed meaningful trends. This reinforces the idea that COD performance is less influenced by general resistance training and more dependent on sprint-specific and technical stimuli.

## Limitations

While the results of the present study offer valuable insights, several limitations must be addressed. First, the a priori sample size calculation was based on a large effect size (d = 1.58) derived from vertical jump height data in a comparable population, which may have resulted in an optimistic sample size estimate. The study may therefore have been underpowered to detect smaller effects, particularly for outcomes with more modest expected changes. Second, a meaningful dropout during the follow-up phase created imbalances between groups, which could potentially introduce bias and limit the assessment of the detraining effects; as reflected in some baseline imbalances in the Treatment coefficients observed in the detraining-phase analyses (Table S2, available online), which should be considered when interpreting these results. Third, while the EBT protocol was standardized, external variables such as field training and match intensity were not controlled. To address this limitation, future studies should incorporate monitoring devices, such as GPS, to assist in controlling such variables. Fourth, the training program did not include exercises that targeted the quadriceps directly, which may have limited the observed KE gains in the U15 players. Future investigations should include exercises that are more quadricep-focused, possibly enhancing training effectiveness and offer a more accurate assessment of KE strength adaptations across these age groups. Fifth, the CG was not time- or attention-matched to the EG, since players in the control condition did not receive any structured intervention during the study period. Future studies should consider incorporating these control condition to better isolate the effects of the intervention from those of increased supervision and structured engagement. Sixth, blinding of participants and the investigators delivering the intervention was not possible given the nature of the EBT protocol, which is an inherent limitation of this type of exercise-based intervention. Seventh, although randomization provided support for its plausibility, the parallel trends assumption from the DiD framework was not empirically testable given the single preintervention measurement. Future studies would benefit from incorporating multiple baseline assessments before the intervention period, which would allow empirical verification of this assumption and strengthen confidence in the causal interpretation of the observed effects. Finally, the study population only consisted of amateur female soccer players, so the findings should not be generalized to populations such as male players, elite players, or even players from different sports. Future research should attempt to replicate this protocol in different categories of players with various training backgrounds, to cross-validate and generalize the current results.

## Conclusion

This study determined that a 9-week EBT program increased lower-limb strength among trained and highly trained female soccer players, particularly in KF across all age groups, and KE in U19 and Senior athletes. In contrast, vertical jump and COD speed performance showed minimal changes. After a 3-week detraining period, most strength gains declined, emphasizing the need for continuous training to preserve neuromuscular adaptations. The findings of this study offer useful practical applications for coaches and strength and conditioning professionals working in female soccer. EBT offers an effective and accessible way of lower-limb strength development, particularly in settings where there is limited access to traditional gym equipment.

## Supplemental Material

sj-docx-1-sph-10.1177_19417381261490002 – Supplemental material for Elastic Band Training Across Competitive Levels in Female Soccer: A Randomized Controlled Trial With Follow-up Supplemental material, sj-docx-1-sph-10.1177_19417381261490002 for Elastic Band Training Across Competitive Levels in Female Soccer: A Randomized Controlled Trial With Follow-up by Henrique Sousa, Eduardo Abade, Júlio A. Costa, João Barreira, Aarón Miralles-Iborra, Filipe Maia and Rui Marcelino in Sports Health

## Data Availability

The data for this study are available from the corresponding author upon reasonable request.
