# Supplementary material for: Elastic Band Training Across Competitive Levels in Female Soccer: A Randomized Controlled Trial With Follow-up
Source: Sports Health. 2026 Oct 12:19417381261490002. Online ahead of print. doi: 10.1177/19417381261490002 (PMC13661821; doi:10.1177/19417381261490002)
Supplement: Supplementary file 1 [file sj-docx-1-sph-10.1177_19417381261490002.docx]

**Effects of In-Season Elastic Band Resistance Training on Physical Performance in Female Soccer Players Across Competitive Levels: A Randomized Controlled Trial with Follow-Up**

**Supplementary Material**

**Contents:**

**Table S1 -** Key outcomes from DiD OLS regression models analysing EBT effects on players from U15, U19 and Senior players following the 9-week protocol.

**Table S2 -** Key outcomes from DiD OLS regression models analysing the detraining effects observed after the 3-week washout period following the 9-week protocol.

**Appendix A -** Elastic band resistance training exercise instructions and order.

**Explanation of statistical terms and abbreviations used in OLS regression output**

Table S1 and S2 report the estimated coefficients (Coef.) together with their standard errors (SE), z-values, and corresponding p-values and 95% confidence intervals (95%CI). The ‘Intercept’ represents the baseline value of the dependent variable when all predictors are set to zero. The ‘Treatment’ coefficient reflects the average effect of being in the experimental group compared with the control group, regardless of time. The ‘Period’ coefficient captures the average difference between pre- and post-intervention measurements across all players. Finally, the ‘DiD’ term represents the effect of the intervention, after accounting for both group and time differences, and is therefore the primary parameter of interest in this study.

| **Table S1 –** Key outcomes from DiD OLS regression models analysing EBT effects on players from U15, U19 and Senior players following the 9-week protocol. | | | | | | | | | | | | | | | | | | | |
| --- | --- | --- | --- | --- | --- | --- | --- | --- | --- | --- | --- | --- | --- | --- | --- | --- | --- | --- | --- |
|  |  | **U15 (n=25)** | | | | | | **U19 (n=25)** | | | | | | **Senior (n=24)** | | | | | |
| **Metrics** | **Variable** | **Coef.** | **SE** | **z-value** | ***p*-value** | **95%CI** | **Sig.** | **Coef.** | **SE** | **z-value** | ***p*-value** | **95%CI** | **Sig.** | **Coef.** | **SE** | **z-value** | ***p*-value** | **95%CI** | **Sig.** |
| **KEND (N)** | Intercept | 282.75 | 29.33 | 9.64 | 0.00 | [225.26, 340.24] |  | 339.00 | 44.39 | 7.64 | 0.00 | [252.00, 426.00] |  | 378.83 | 56.44 | 6.71 | 0.00 | [268.21, 489.45] |  |
|  | Treatment | 61.40 | 35.29 | 1.74 | 0.08 | [-7.77, 130.57] | * | -33.77 | 50.91 | -0.66 | 0.51 | [-133.55, 66.01] |  | -9.33 | 71.57 | -0.13 | 0.90 | [-149.61, 130.95] |  |
|  | Period | 39.33 | 40.00 | 0.98 | 0.33 | [-39.07, 117.73] |  | 19.33 | 50.37 | 0.38 | 0.70 | [-79.40, 118.06] |  | 20.83 | 72.53 | 0.29 | 0.77 | [-121.33, 162.99] |  |
|  | DiD | 1.28 | 54.64 | 0.02 | 0.98 | [-105.81, 108.37] |  | 131.59 | 62.05 | 2.12 | 0.03 | [9.97, 253.21] | ** | 87.33 | 99.31 | 0.88 | 0.38 | [-107.32, 281.98] |  |
| **KED (N)** | Intercept | 295.25 | 35.37 | 8.35 | 0.00 | [225.92, 364.58] |  | 339.50 | 46.15 | 7.36 | 0.00 | [249.05, 429.95] |  | 433.17 | 52.28 | 8.29 | 0.00 | [330.70, 535.64] |  |
|  | Treatment | 67.37 | 41.73 | 1.61 | 0.11 | [-14.42, 149.16] |  | 5.81 | 57.10 | 0.10 | 0.92 | [-106.11, 117.73] |  | -46.92 | 69.29 | -0.68 | 0.50 | [-182.73, 88.89] |  |
|  | Period | 19.08 | 45.47 | 0.42 | 0.66 | [-70.04, 108.20] |  | 20.17 | 51.49 | 0.39 | 0.70 | [-80.75, 121.09] |  | 24.58 | 73.32 | 0.34 | 0.74 | [-119.13, 168.29] |  |
|  | DiD | 25.92 | 60.33 | 0.43 | 0.67 | [-92.33, 144.17] |  | 132.30 | 66.88 | 1.99 | 0.05 | [1.22, 263.38] | ** | 95.75 | 103.25 | 0.93 | 0.35 | [-106.62, 298.12] |  |
| **KFND (N)** | Intercept | 182.83 | 15.67 | 11.67 | 0.00 | [152.12, 213.54] |  | 186.08 | 16.03 | 11.61 | 0.00 | [154.66, 217.50] |  | 199.33 | 15.76 | 12.65 | 0.00 | [168.44, 230.22] |  |
|  | Treatment | -26.83 | 19.51 | -1.38 | 0.17 | [-65.07, 11.41] |  | 32.76 | 20.16 | 1.63 | 0.10 | [-6.75, 72.27] | * | -7.92 | 22.28 | -0.36 | 0.72 | [-51.59, 35.75] |  |
|  | Period | -19.92 | 18.00 | -1.11 | 0.27 | [-55.20, 15.36] |  | -9.92 | 21.29 | -0.47 | 0.64 | [-51.65, 31.81] |  | -6.00 | 23.35 | -0.26 | 0.80 | [-51.77, 39.77] |  |
|  | DiD | 48.53 | 24.49 | 1.98 | 0.05 | [0.53, 96.53] | ** | 29.84 | 28.39 | 1.05 | 0.29 | [-25.80, 85.48] |  | 61.50 | 33.62 | 1.83 | 0.07 | [-4.40, 127.40] | * |
| **KFD (N)** | Intercept | 182.33 | 11.99 | 15.20 | 0.00 | [158.83, 205.83] |  | 205.42 | 15.05 | 13.65 | 0.00 | [175.92, 234.92] |  | 215.17 | 18.94 | 11.36 | 0.00 | [178.05, 252.29] |  |
|  | Treatment | -26.87 | 14.31 | -1.88 | 0.06 | [-54.92, 1.18] | * | 33.05 | 22.99 | 1.44 | 0.15 | [-12.01, 78.11] |  | -27.25 | 21.94 | -1.24 | 0.21 | [-70.25, 15.75] |  |
|  | Period | 2.42 | 14.98 | 0.16 | 0.87 | [-26.94, 31.78] |  | -23.92 | 20.19 | -1.19 | 0.24 | [-63.49, 15.65] |  | -15.92 | 26.62 | -0.60 | 0.55 | [-68.10, 36.26] |  |
|  | DiD | 37.74 | 20.36 | 1.85 | 0.06 | [-2.17, 77.65] | * | 34.38 | 33.60 | 1.02 | 0.31 | [-31.48, 100.24] |  | 56.67 | 33.71 | 1.68 | 0.09 | [-9.40, 122.74] | * |
| **CMJ (cm)** | Intercept | 21.44 | 1.47 | 14.62 | 0.00 | [18.56, 24.32] |  | 21.64 | 0.60 | 36.23 | 0.00 | [20.46, 22.82] |  | 22.79 | 1.42 | 16.08 | 0.00 | [20.01, 25.57] |  |
|  | Treatment | 0.37 | 1.69 | 0.22 | 0.83 | [-2.94, 3.68] |  | -1.12 | 1.20 | -0.94 | 0.34 | [-3.47, 1.23] |  | -2.45 | 1.66 | -1.48 | 0.14 | [-5.70, 0.80] |  |
|  | Period | -0.23 | 1.97 | -0.12 | 0.91 | [-4.09, 3.63] |  | -0.04 | 0.87 | -0.05 | 0.96 | [-1.75, 1.67] |  | 0.53 | 1.90 | 0.28 | 0.78 | [-3.19, 4.25] |  |
|  | DiD | 2.07 | 2.34 | 0.89 | 0.38 | [-2.52, 6.66] |  | 1.01 | 1.59 | 0.64 | 0.53 | [-2.11, 4.13] |  | 1.83 | 2.31 | 0.79 | 0.43 | [-2.70, 6.36] |  |
| **CMJ (W)** | Intercept | 2166.58 | 100.53 | 21.55 | 0.00 | [1969.54, 2363.62] |  | 2255.33 | 135.83 | 16.60 | 0.00 | [1989.10, 2521.56] |  | 2659.50 | 188.13 | 14.14 | 0.00 | [2290.77, 3028.23] |  |
|  | Treatment | 48.34 | 195.07 | 0.25 | 0.80 | [-334.00, 430.68] |  | 24.59 | 159.20 | 0.15 | 0.88 | [-287.44, 336.62] |  | -115.58 | 229.85 | -0.50 | 0.62 | [-566.09, 334.93] |  |
|  | Period | 214.75 | 191.59 | 1.12 | 0.26 | [-160.77, 590.27] |  | 210.08 | 274.54 | 0.77 | 0.44 | [-328.02, 748.18] |  | -157.50 | 278.89 | -0.57 | 0.57 | [-704.12, 389.12] |  |
|  | DiD | 87.10 | 344.60 | 0.25 | 0.80 | [-588.32, 762.52] |  | 135.46 | 337.23 | 0.40 | 0.69 | [-525.51, 796.43] |  | 581.08 | 346.41 | 1.68 | 0.09 | [-97.88, 1260.04] | * |
| **T-Test (s)** | Intercept | 12.48 | 0.28 | 44.41 | 0.00 | [11.93, 13.03] |  | 11.97 | 0.15 | 77.74 | 0.00 | [11.68, 12.26] |  | 11.82 | 0.15 | 80.34 | 0.00 | [11.53, 12.11] |  |
|  | Treatment | 0.32 | 0.40 | 0.81 | 0.42 | [-0.46, 1.10] |  | -0.04 | 0.19 | -0.20 | 0.84 | [-0.41, 0.33] |  | 0.10 | 0.27 | 0.39 | 0.70 | [-0.43, 0.63] |  |
|  | Period | 0.28 | 0.41 | 0.68 | 0.50 | [-0.52, 1.08] |  | 0.16 | 0.24 | 0.67 | 0.50 | [-0.31, 0.63] |  | 0.23 | 0.25 | 0.94 | 0.35 | [-0.26, 0.72] |  |
|  | DiD | -0.02 | 0.56 | -0.01 | 0.97 | [-1.12, 1.08] |  | -0.13 | 0.34 | -0.38 | 0.70 | [-0.80, 0.54] |  | 0.02 | 0.46 | 0.05 | 0.96 | [-0.88, 0.92] |  |

**KEND:** knee extension of the non-dominant leg; **KED:** knee extension of the dominant leg; **KFND:** knee flexion of the non-dominant leg; **KFD:** knee flexion of the dominant leg; **CMJ:** countermovement jump; **N:** newtons; **cm:** centimetres; **W:** watts; **s:** seconds; **Intercept**: control group´s average score at baseline; **Treatment:** baseline difference between experimental and control group; **Period:** overall change over time for the control group; **DiD:** change over time in the experimental group compared to the control group; **Coef.:** coefficient; **SE:** standard error; *p≤0.10; **p≤0.05; ***p≤0.01

| **Table S2 –** Key outcomes from DiD OLS regression models analysing the detraining effects observed after the 3-week washout period following the 9-week protocol. | | | | | | | | | | | | | | | | | | | |
| --- | --- | --- | --- | --- | --- | --- | --- | --- | --- | --- | --- | --- | --- | --- | --- | --- | --- | --- | --- |
|  |  | **U15 (n=23)** | | | | | | **U19 (n=22)** | | | | | | **Senior (n=21)** | | | | | |
| **Metrics** | **Variable** | **Coef.** | **SE** | **z-value** | ***p*-value** | **95%CI** | **Sig.** | **Coef.** | **SE** | **z-value** | ***p*-value** | **95%CI** | **Sig.** | **Coef.** | **SE** | **z-value** | ***p*-value** | **95%CI** | **Sig.** |
| **KEND (N)** | Intercept | 307.20 | 24.37 | 12.61 | 0.00 | [259.43, 354.97] |  | 367.20 | 23.68 | 15.51 | 0.00 | [320.79, 413.61] |  | 438.40 | 44.10 | 9.94 | 0.00 | [351.96, 524.84] |  |
|  | Treatment | 77.57 | 39.93 | 1.94 | 0.05 | [-0.69, 155.83] | * | 96.05 | 36.31 | 2.65 | 0.01 | [24.88, 167.22] | *** | 58.78 | 68.30 | 0.86 | 0.39 | [-75.09, 192.65] |  |
|  | Period | 29.50 | 56.10 | 0.53 | 0.60 | [-80.46, 139.46] |  | -26.70 | 44.91 | -0.59 | 0.55 | [-114.72, 61.32] |  | -32.20 | 76.67 | -0.42 | 0.67 | [-182.47, 118.07] |  |
|  | DiD | -52.27 | 72.23 | -0.72 | 0.47 | [-193.84, 89.30] |  | -90.97 | 64.58 | -1.41 | 0.16 | [-217.55, 35.61] |  | -40.53 | 107.15 | -0.38 | 0.71 | [-250.54, 169.48] |  |
| **KED (N)** | Intercept | 312.80 | 33.43 | 9.36 | 0.00 | [247.28, 378.32] |  | 364.70 | 20.32 | 17.95 | 0.00 | [324.87, 404.53] |  | 502.10 | 49.50 | 10.14 | 0.00 | [405.08, 599.12] |  |
|  | Treatment | 94.82 | 46.89 | 2.02 | 0.04 | [2.92, 186.72] | ** | 136.22 | 34.98 | 3.89 | 0.00 | [67.66, 204.78] | *** | 26.17 | 75.65 | 0.35 | 0.73 | [-122.10, 174.44] |  |
|  | Period | -12.00 | 54.96 | -0.22 | 0.83 | [-119.72, 95.72] |  | -21.30 | 46.06 | -0.46 | 0.64 | [-111.58, 68.98] |  | -46.20 | 83.01 | -0.56 | 0.58 | [-208.90, 116.50] |  |
|  | DiD | -66.46 | 69.37 | -0.96 | 0.23 | [-202.43, 69.51] |  | -117.70 | 65.18 | -1.80 | 0.07 | [-245.45, 10.05] | * | -63.80 | 110.84 | -0.58 | 0.57 | [-281.05, 153.45] |  |
| **KFND (N)** | Intercept | 158.30 | 9.93 | 15.94 | 0.00 | [138.84, 177.76] |  | 173.30 | 8.46 | 20.48 | 0.00 | [156.72, 189.88] |  | 201.80 | 17.84 | 11.31 | 0.00 | [166.83, 236.77] |  |
|  | Treatment | 26.32 | 15.46 | 1.70 | 0.09 | [-3.98, 56.62] | * | 64.62 | 17.68 | 3.66 | 0.00 | [29.97, 99.27] | *** | 53.02 | 25.36 | 2.09 | 0.04 | [3.31, 102.73] | ** |
|  | Period | 29.00 | 12.75 | 2.28 | 0.02 | [4.01, 53.99] | ** | 9.60 | 13.60 | 0.71 | 0.48 | [-17.06, 36.26] |  | 15.40 | 24.54 | 0.63 | 0.53 | [-32.70, 63.50] |  |
|  | DiD | -34.31 | 23.55 | -1.46 | 0.15 | [-80.47, 11.85] |  | -23.85 | 23.06 | -1.03 | 0.30 | [-69.05, 21.35] |  | -39.95 | 34.38 | -1.16 | 0.25 | [-107.33, 27.43] |  |
| **KFD (N)** | Intercept | 180.60 | 10.09 | 17.90 | 0.00 | [160.82, 200.38] |  | 180.90 | 11.04 | 16.38 | 0.00 | [159.26, 202.54] |  | 211.20 | 19.41 | 10.88 | 0.00 | [173.16, 249.24] |  |
|  | Treatment | 15.02 | 15.18 | 0.99 | 0.32 | [-14.73, 44.77] |  | 61.93 | 23.94 | 2.59 | 0.01 | [15.01, 108.85] | *** | 26.35 | 25.33 | 1.04 | 0.30 | [-23.30, 76.00] |  |
|  | Period | 10.50 | 16.17 | 0.65 | 0.52 | [-21.19, 42.19] |  | 16.30 | 13.62 | 1.20 | 0.23 | [-10.40, 43.00] |  | 18.60 | 25.73 | 0.72 | 0.47 | [-31.83, 69.03] |  |
|  | DiD | -24.88 | 24.32 | -1.02 | 0.31 | [-72.55, 22.79] |  | -30.80 | 31.10 | -0.99 | 0.32 | [-91.76, 30.16] |  | -34.87 | 33.56 | -1.04 | 0.30 | [-100.65, 30.91] |  |
| **CMJ (cm)** | Intercept | 22.01 | 1.37 | 16.09 | 0.00 | [19.32, 24.70] |  | 21.49 | 0.53 | 40.60 | 0.00 | [20.45, 22.53] |  | 23.41 | 1.20 | 19.49 | 0.00 | [21.06, 25.76] |  |
|  | Treatment | 1.64 | 1.67 | 0.99 | 0.32 | [-1.63, 4.91] |  | 0.44 | 0.93 | 0.48 | 0.64 | [-1.38, 2.26] |  | -0.59 | 1.62 | -0.37 | 0.72 | [-3.77, 2.59] |  |
|  | Period | 0.28 | 1.93 | 0.15 | 0.89 | [-3.50, 4.06] |  | 0.94 | 0.79 | 1.20 | 0.23 | [-0.61, 2.49] |  | -0.08 | 1.90 | -0.04 | 0.97 | [-3.80, 3.64] |  |
|  | DiD | -1.90 | 2.33 | -0.82 | 0.41 | [-6.47, 2.67] |  | -1.22 | 1.53 | -0.80 | 0.42 | [-4.22, 1.78] |  | -1.37 | 2.46 | -0.56 | 0.58 | [-6.19, 3.45] |  |
| **CMJ (W)** | Intercept | 2410.40 | 189.40 | 12.73 | 0.00 | [2039.18, 2781.62] |  | 2366.70 | 204.99 | 11.55 | 0.00 | [1964.92, 2768.48] |  | 2616.50 | 230.55 | 11.35 | 0.00 | [2164.62, 3068.38] |  |
|  | Treatment | 106.37 | 299.96 | 0.36 | 0.72 | [-481.55, 694.29] |  | 255.47 | 281.85 | 0.91 | 0.37 | [-296.96, 807.90] |  | 387.86 | 285.21 | 1.36 | 0.17 | [-171.15, 946.87] |  |
|  | Period | -34.40 | 201.41 | -0.17 | 0.86 | [-429.16, 360.36] |  | 407.40 | 328.54 | 1.24 | 0.22 | [-236.54, 1051.34] |  | 38.30 | 303.58 | 0.13 | 0.90 | [-556.72, 633.32] |  |
|  | DiD | -157.60 | 388.96 | -0.41 | 0.69 | [-919.96, 604.76] |  | -405.15 | 421.99 | -0.96 | 0.34 | [-1232.25, 421.95] |  | -95.39 | 414.91 | -0.23 | 0.82 | [-908.61, 717.83] |  |
| **T-Test (s)** | Intercept | 12.71 | 0.34 | 37.26 | 0.00 | [12.04, 13.38] |  | 12.23 | 0.20 | 61.33 | 0.00 | [11.84, 12.62] |  | 12.01 | 0.19 | 62.48 | 0.00 | [11.64, 12.38] |  |
|  | Treatment | 0.39 | 0.40 | 0.95 | 0.34 | [-0.39, 1.17] |  | -0.29 | 0.31 | -0.95 | 0.34 | [-0.90, 0.32] |  | 0.20 | 0.40 | 0.50 | 0.62 | [-0.58, 0.98] |  |
|  | Period | -0.09 | 0.44 | -0.19 | 0.85 | [-0.95, 0.77] |  | -0.25 | 0.23 | -1.10 | 0.27 | [-0.70, 0.20] |  | -0.07 | 0.29 | -0.24 | 0.81 | [-0.64, 0.50] |  |
|  | DiD | 0.28 | 0.59 | 0.47 | 0.64 | [-0.88, 1.44] |  | 0.24 | 0.38 | 0.62 | 0.54 | [-0.50, 0.98] |  | 0.05 | 0.57 | 0.09 | 0.93 | [-1.07, 1.17] |  |

**KEND:** knee extension of the non-dominant leg; **KED:** knee extension of the dominant leg; **KFND:** knee flexion of the non-dominant leg; **KFD:** knee flexion of the dominant leg; **CMJ:** countermovement jump; **N:** newtons; **cm:** centimetres; **W:** watts; **s:** seconds; **Intercept**: control group´s average score at baseline; **Treatment:** baseline difference between experimental and control group; **Period:** overall change over time for the control group; **DiD:** change over time in the experimental group compared to the control group; **Coef.:** coefficient; **SE:** standard error; *p≤0.10; **p≤0.05; ***p≤0.01

| **Appendix A -** Elastic band resistance training exercise instructions and order. | | | | |  |
| --- | --- | --- | --- | --- | --- |
| **EBT1** | | | | |  |
| **Glute Bridges / Hip Thrust**  **3 x 8-10 reps** | |  | **Place the band around the hips and secure it to the floor with your feet.**  **Starting Position:** Lie on your back with your knees bent and feet flat on the floor. Feet should be hip-width apart, with toes pointing forward.  **Concentric Movement:** Squeeze the glutes and abdominals and lift the hips. Raise the hips until the body forms a straight line from the knees to the hips and shoulders.  **Eccentric Movement:** Slowly lower the hips back down to the floor, maintaining tension in the glutes and abdominals. | |  |
| **Hip Abduction**  **3 x 8-10 reps** | |  | **Starting Position:** The legs should be positioned as far apart as the minimum resistance of the band allows. **Concentric Movement:** Raise the legs as quickly as possible against the resistance of the band, keeping the legs straight and toes pointing upward. Lift until a strong contraction is felt in the abductors.  **Eccentric Movement:** Lower the legs back to the starting position in a controlled manner (~ 3 seconds), maintaining tension in the resistance band. | |  |
| **Hip Adduction**  **3 x 8-10 reps** | |  | **The resistance band is secured between the feet of a second person.**  **Starting Position:** The lower leg must remain straight, with minimal tension felt in the resistance band.  **Concentric Movement:** Raise the leg as quickly as possible against the resistance of the band, keeping the leg straight and the toes pointing upward. Lift until a strong contraction is felt in the adductors.  **Eccentric Movement:** Lower the leg back to the starting position in a controlled manner (~ 3 seconds), maintaining tension in the resistance band. | |  |
|  | **EBT2** | | | | |
|  | **Romanian**  **Deadlift**  **3 x 8-10 reps** |  | | **Starting Position:** Stand upright with the chest lifted and the shoulders, back, and core engaged.  **Eccentric Movement:** Keep the back straight and maintain a slight bend in the knees. In a controlled manner (~ 3 seconds), lower the torso toward the ground, bringing the band closer to the feet. Lower until the torso is parallel to the floor.  **Concentric Movement:** Keep the back straight throughout the entire movement. Drive the hips forward to stand up as quickly as possible, squeezing the glutes at the top. | |
|  | **Hip Flexion**  **3 x 8-10 reps** |  | | **The resistance band is attached to one ankle of a second person or to a fixed point (e.g., goalpost).**  **Starting Position:** Stand with feet hip-width apart, engage the abdominals, and ensure the back remains straight.  **Concentric Movement:** Lift the leg forward with the knee straight, working against the resistance of the band. Raise the leg until a strong contraction is felt in the quadriceps.  **Eccentric Movement:** In a controlled manner (~3 seconds), slowly return to the starting position, resisting the pull of the band. | |
|  | **Trunk Rotation**  **3 x 8-10 reps** |  | | **The resistance band is attached to a fixed point (e.g., goalpost).**  **Starting Position:** Fully extend the arms forward, holding the band with both hands. Position the body away from the anchor point to ensure adequate resistance.  **Concentric Movement:** Rotate the torso away from the anchor point, pulling the band across the body. Keep the arms fully extended and perform the movement as quickly as possible.  **Eccentric Movement:** In a controlled manner (~3 seconds), slowly return to the starting position, resisting the pull of the band. | |
